# Comments on letter to the editor by Faniyan *et al*. in response to Imported leishmaniasis in Sweden 1993–2016

**DOI:** 10.1017/S0950268818002959

**Published:** 2018-11-16

**Authors:** S. K. Söbirk, M. Inghammar, M. Collin, L. Davidsson

**Affiliations:** 1 Division of Infection Medicine, Department of Clinical Sciences, Lund University, Lund, Sweden; 2 Unit for Parasitology and Waterborne Diseases, Public Health Agency of Sweden, Solna, Sweden

We are pleased that Faniyan *et al*. found our article helpful. The aim of our study was to estimate the incidence of imported leishmaniasis in Sweden and to describe the clinical presentation, patient characteristics, country where the infection was acquired and causative species. This is the first nationwide, epidemiological study from Sweden published to date. Unlike the United States, there is no risk for transmission of leishmaniasis in Sweden, as neither the sand fly vector nor infected animal reservoir occurs here [1].

We acknowledged that the true incidence of leishmaniasis is probably higher than our estimation from retrospectively collected data, as our data excluded individuals who did not seek healthcare or where the clinician did not suspect leishmaniasis.

The increased proportion of children in the group of confirmed cases during the last four years (2012–2016) reflects the proportion of children in the groups immigrating to Sweden from endemic countries. In 2015, the number of applicants for asylum reached an historical peak in Sweden. The majority of asylum seekers were from Syria and Afghanistan, and 51% of these were children under 18 years of age [2].

As all cases of leishmaniasis in Sweden are imported, variations in our estimated incidence reflect patterns of migration and travel to and from endemic countries. These variations do not reflect socioeconomic factors, zoonotic seroprevalence, environmental or climate change in Sweden. Although very relevant for incidence of leishmaniasis worldwide, the complex interplay between the above-mentioned factors lies outside the scope of our study. The rise in incidence over the last few years of the study reflects a greater immigration of people from endemic areas compared to previous years [3, 4]. We hope to raise awareness of this rare imported disease amongst healthcare providers in a non-endemic setting, so that patients infected with *Leishmania* will receive a correct diagnosis and the appropriate treatment.
